#  Monitoring cardiovascular disease risk in children with cystic fibrosis using arterial stiffness: A new perspective 

**DOI:** 10.5578/tt.202501999

**Published:** 2025-03-24

**Authors:** Gökçen KARTAL ÖZTÜRK, Aykut EŞKİ, Figen ÇELEBİ ÇELİK, Seçil CONKAR, Ahmet KESKİNOĞLU, Figen GÜLEN, Esen DEMİR

**Affiliations:** 1 Division of Pediatric Pulmonology, Department of Child Health and Diseases, Ege University Faculty of Medicine, İzmir, Türkiye; 2 Clinic of Pediatric Pulmonology, University of Health Sciences, Tepecik Education and Research Hospital, İzmir, Türkiye; 3 Clinic of Pediatric Allergy, University of Health Sciences, Dr. Behçet Uz Children’s Hospital, İzmir, Türkiye; 4 Division of Pediatric Nephrology, Department of Child Health and Diseases, Ege University Faculty of Medicine, İzmir, Türkiye

## Abstract

**ABSTRACT**

** Monitoring cardiovascular disease risk in children with cystic fibrosis using arterial stiffness: A new perspective **

**Introduction:** *Early diagnosis with newborn screening programs and prolonged life expectancy with new treatment strategies have made cardiovascular disease (CVD) one of the important issues in cystic fibrosis (CF). In the early stages of CVD, it is difficult to recognize and follow-up increased arterial stiffness with conventional methods. Different measurement methods are needed. Therefore, in this study, we aimed to use arterial stiffness measurements in the follow-up of children with CF. *

**Materials and Methods:** *This is a follow-up study examining the changes in arterial stiffness in children with CF by repeating hemodynamic measurements [augmentation index (AIx) and pulse wave velocity (PWV)]. We repeated hemodynamic measurements and CF-related CVD risk factors (Atherosclerosis risk factors: Fasting blood sugar, lipid profiles, and HbA1c) and systemic inflammation markers [C-reactive protein (CRP) and immunoglobulin G and pulmonary function tests] in children undergoing routine annual complication evaluation and examined changes during follow-up. *

**Results:** *Hemodynamic measurements could be repeated in 37 of 52 patients due to inclusion criteria. Mean age of the study group was 12 ± 4.5 years and 48.6% were female. There was a statistically significant increase in high density lipoprotein, HbA1c, and CRP and a decrease in low density lipoprotein and FEV1 at the follow-up. Heart rate, central blood pressure, augmented pressure, and PWV were similar. AIx, peripheral systolic blood pressure (SBP), and mean arterial pressure were increased significantly (p < 0.05). The increase in AIx was greater than expected for age and greater in female patients and in those with low body mass index, moderate-severe disease, and high CRP levels. Also, the change in AIx was positively correlated with changes in peripheral SBP and CRP. *

**Conclusion:** *This is the first study to evaluate the use of PWV and AIx in the follow-up of children with CF and showed that arterial stiffness measured with AIx increased at follow-up. The use of markers of arterial stiffness in CF from childhood onwards may enable early detection and monitoring of CVD risk and future prevention. *

**Key words:** *Arterial stiffness; augmentation index; cystic fibrosis; pulse wave velocity analysis *

**ÖZ**

** Kistik fibrozisli çocuklarda kardiyovasküler hastalık riskinin arteriyel sertlik ile izlenmesi: Yeni bir bakış açısı **

**Giriş:** *Yenidoğan tarama programları ile erken tanı konulması ve yeni tedavi stratejileri ile uzamış yaşam süresi kardiyovasküler hastalığı (KVH) kistik fibroziste (KF) dikkat çeken konulardan biri haline getirmiştir. Kardiyovasküler hastalıkların erken evrelerinde arteriyel sertlik artar ve bu evrede ekokardiyografi gibi konvansiyonel yöntemlerle değişiklikleri tanımak ve takip etmek zordur. Farklı ölçüm metotlarına ihtiyaç duyulmaktadır. Bu nedenle, bu çalışmada KF’li çocukların takibinde arteriyel sertlik ölçümlerini kullanmayı amaçladık. *

**Materyal ve Metod:** *Kistik fibrozisli çocuklarda arteriyel sertliğin hemodinamik ölçümler [augmentasyon indeksi (Aİ) ve nabız dalga hızı (PWV)] tekrarlanarak değerlendirildiği bir takip çalışmasıdır. Yıllık rutin komplikasyon değerlendirmesi yapılan çocuklarda hemodinamik ölçümleri ve KF ilişkili KVH risk faktörlerini (Ateroskleroz risk faktörleri: Açlık kan şekeri, lipid profili, HbA1c), sistemik inflamasyon belirteçleri [C-reaktif protein (CRP) ve immünoglobulin G ve solunum fonksiyon testleri] tekrarladık ve takipteki değişimlerini inceledik. *

**Bulgular:** *Hemodinamik ölçümler dahil edilme kriterleri nedeniyle 52 hastanın 37’sinde tekrarlanabilmiştir. Çalışma grubunun yaş ortalaması 12 ± 4.5 yıl olup %48.6’sı kadındı. Takipte yüksek yoğunluklu lipoprotein, HbA1c ve CRP’de istatistiksel olarak anlamlı bir artış, düşük yoğunluklu lipoprotein ve FEV1’de ise azalma görülmüştür. Kalp hızı, merkezi kan basıncı, artmış basınç ve PWV takipte benzerdi (p > 0.05). Augmentasyon indeksi, ortalama arter basıncı ve periferik sistolik kan basıncı (SKB) anlamlı olarak artmıştı (p < 0.05). Augmentasyon indeksindeki artış yaşa göre beklenenden daha büyüktü ve kadınlarda; düşük vücut kitle indeksi, orta-şiddetli hastalık ve yüksek CRP düzeyleri olan hastalarda daha fazlaydı. Ayrıca, Aİ’deki değişim periferik SKB ve CRP’deki değişimlerle pozitif korelasyon gösterdi. *

**Sonuç:** *Bu çalışma, KF’li çocukların takibinde KVH risk belirteçleri PWV ve Aİ’nin kullanımını değerlendiren ilk çalışmadır ve KF’li çocuklarda Aİ ile ölçülen arteriyel sertliğin takipte arttığını göstermiştir. Çocukluk çağından itibaren KF’te arteriyel sertlik belirteçlerinin kullanımı, KVH riskinin erken saptanmasını, takibini ve gelecekte önlemler alınmasını sağlayabilir. *

**Anahtar kelimeler:** *Arteriyel sertlik; augmentasyon indeksi; kistik fibrozis; nabız dalga hızı analizi *

## INTRODUCTION

Cystic fibrosis (CF) is an autosomal recessive disorder characterized by the absence or dysfunction of the CF transmembrane conductance regulator (CFTR) pro- tein due to mutations in the CFTR gene. As a result of CFTR dysfunction, disease occurs in the respiratory, gastrointestinal, endocrine, and reproductive systems

(1). Cardiovascular disease (CVD) may develop sec- ondary to CF-related conditions such as progressive lung damage, oxidative stress, inflammation, diabe- tes, etc. Moreover, the localization of CFTR in vascu- lar endothelial and smooth muscle cells can cause cardiovascular complications with microvascular dysfunction (2-5). As a result of early diagnoses with newborn screening programs and increased lifespan with new treatment strategies, altered body composi- tion with increased fat absorption and body mass index (BMI), and hypertension suggest that more attention should be paid to CVD in these patients.

In the early process of CVD, arterial stiffness increases, and at this stage, it is difficult to recognize the vascular changes in patients with conventional

methods such as echocardiography. In recent years, pulse wave velocity (PWV) and augmentation index (AIx) have frequently been used to evaluate CVD risk by measuring arterial stiffness in many diseases (6,7). In a study with adult CF patients, AIx has been found to be elevated in patients compared to healthy groups and increased with aging and the presence of CF-related diabetes (CFRD) (8). Also, our previous study in children with CF showed that increased arterial stiffness (increased AIx) and vascular changes begin in childhood despite the absence of traditional risk factors [increased low density lipoprotein (LDL) and cholesterol, hypertension, and obesity] (9).

Practice guidelines do not discuss routine CVD mon- itoring recommendations for the CF population, and there is limited research on this condition (10). Therefore, more longitudinal follow-up studies are needed to design CF-related CVD-specific screening guidelines. Based on this shortcoming, we hypothe- sized that PWV and AIx could be used for CVD risk assessment and follow-up in CF patients due to non-invasive, easy, and reproducible tests. Therefore,

as a longitudinal study of our previous study, we aimed to examine the change in arterial stiffness measurements and CF-specific risk factors in the fol- low-up of CF and to evaluate whether these measure- ments can be used in the follow-up of CF patients. It was hypothesized that arterial stiffness increases with age and CF-specific risk factors in children with CF. Recognizing and monitoring CVD and risk factors in CF may allow the development of new follow-up and treatment strategies.

### MATERIALS and METHODS

**Study Design**

This is a longitudinal study in which arterial stiffness was monitored by measuring PWV and AIx in chil- dren with CF at our university’s Pediatric Pulmonology and Pediatric Nephrology departments. All subjects provided written informed consent, and the Local Research Ethics Committee approved the study (19- 6.1T/40). We repeated hemodynamic measurements (AIx and PWV) and CF-related CVD risk factors that were performed in our previous study and evaluated their change at about a year later follow-up.

In our unit, patients are routinely evaluated every year for CF complications with anthropometric mea- surements, laboratory tests, pulmonary function tests (PFT), and, if necessary, imaging methods. Patients who had previously undergone hemodynamic mea- surements and completed annual complication eval- uations were invited to the clinic by telephone for repeat measurements. Children over the age of 18 years, with obesity, pulmonary hypertension (PH), CVD, and CFRD, receiving continuous oxygen sup- port, non-invasive mechanical ventilation, and sys- temic steroid therapy were excluded from the study. Children with acute respiratory tract infections in the month before the measurement were also excluded.

Height (cm) and body weight (kg) were measured. BMI was calculated by the kg/cm (m^2^) formula. Atherosclerosis risk factors [fasting blood sugar, lipid profiles (serum total cholesterol, high- and low-den- sity lipoprotein (HDL and LDL) and triglyceride (TG), and HbA1c] and systemic inflammation markers [C-reactive protein (CRP) and immunoglobulin G] were recorded from clinically stable conditions with- in the last month. PFT was performed by spirometry (Flowhandy ZAN 100, Germany) following the American Thoracic Society standards by measuring forced expiratory volume in 1 second (FEV1), forced

vital capacity (FVC), and forced expiratory flow during the middle half of FVC (FEF25‐75) (11).

### Hemodynamic Measurements

Hemodynamic measurements were performed after the patient had rested for 15 minutes, in a sitting position in a quiet room, 12 hours after fasting, and before inhaled treatments such as dornase alpha, short-acting beta-2 agonists, and inhaled antibiotics, by personnel specially trained in the technique and blinded to the clinical characteristics of each patient.

Mobil-O-Graph 24-h PWA Monitor (IEM GmbH, Stolberg, Germany) was used for the measurement. A suitable cuff was selected for brachial artery mea- surement by oscillometric method. A bluetooth con- nection was established between the device and a software program (Hypertension Management System; Client-Server Company, IEM GmbH, Stolberg, Germany) for data analysis. Age, sex, cm, kg, and smoking information were entered into the software program. PWV analysis was performed three times at five-minute intervals. The software program classified the data as high-quality and low-quality, and only high-quality data were evaluated. The calculated PWV was defined as the aortic pulse wave. As the measurement may be affected by heart rate, AIx was normalized to heart rate with 75 beats/min.

### Statistical Analysis

Statistical analysis was performed using IBM SPSS statistics 25.0 (IBM Corp. released 2017. IBM SPSS statistics for Windows, version 25.0. Armonk, NY: IBM Corp.). Numerical variables were presented as median (minimum-maximum) and mean (± standard deviation). The Shapiro-Wilk test was used to test the normal distribution of the numerical variables (n< 50). Categorical variables were presented as num- bers and percentages. Comparisons of continuous paired parameters were made by paired t-test. Correlation analysis between continuous parameters was performed with Pearson’s correlation test. The significance level for all hypotheses was accepted as p< 0.05.

## RESULTS

The study included 52 patients who had previously undergone hemodynamic measurements. Four patients with pulmonary exacerbation in the last month, two patients over the age of 18 years, two with PH and CFRD, two who required respiratory

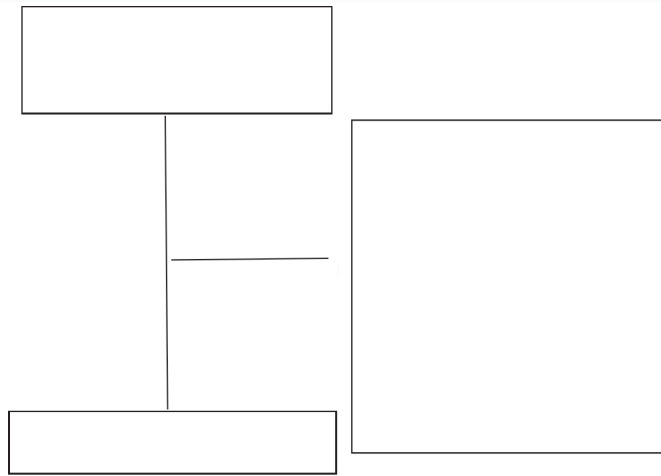
Patients who had previously undergone hemodynamic measurements n= 52

37 children included in the study

Excluded

n= 4, antibiotic use in the last month for pulmonary exacerbation,

n= 2, over the age of 18 years, n= 2, pulmonary hypertension and CF-related diabetes,

n= 2, required respiratory support

n= 2, who died,

n= 3, could not be measured.

**Figure 1.** Flow chart of the study group.

support, two who died, and three patients who could not be measured were excluded. Hemodynamic measurements were repeated in 37 patients (Figure 1). Mean age of the study group was 12 ± 4.5 years and 48.6% were female. There were no patients on CFTR modulator therapy. Clinical characteristics of the study group are shown in Table 1.

**Table:** 

**Table 1.** Clinical characteristics of the study group	
**n= 37**	
Age, years (mean ± SD)	12 ± 4.5
Sex, F/M	18/19
Pancreatic insufficiency, (%)	33 (89.2)
Mutations, (%)	
F508del homozygous	6 (16.2)
F508del heterozygous	13 (35.1)
Others	18 (48.6)
Colonization, (%)	
At first evaluation	22 (59.5)
At second evaluation	29 (78.4)
Microorganism*	
*Staphylococcus aureus*	18/16
*Pseudomonas aeruginosa*	18/24
*Aspergillus* spp.	0/5
Others	0/4
SD: Standard deviation, F/M: Female/Male. *At first evaluation/At second evaluation.

Atherosclerosis risk factors, systemic inflammation markers, PFTs, and hemodynamic measurements could repeated after a median of 14 (12-16) months. BMI, fasting blood glucose, serum total cholesterol, and TG were similar. There was a statistically signifi- cant increase in HDL and HbA1c and a decrease in LDL (p< 0.05). In systemic inflammation markers, while no change was in immunoglobulin G (p> 0.05), there was a significant increase in CRP (p< 0.05). There was a statistically significant decrease in

FEV1 in spirometry (p< 0.05). In hemodynamic mea- surements, heart rate, central blood pressure, aug-

mented pressure, and PWV were similar at follow-up (p> 0.05), AIx, peripheral systolic blood pressure (SBP), and mean arterial pressure were increased significantly (p< 0.05) (Table 2).

The change in AIx was positively correlated with changes in peripheral SBP (r= 0.42 and p< 0.01) and CRP (r= 0.45 and p< 0.01). There was no correlation between the change in AIx and fasting blood glucose, HbA1c, lipid profiles, CRP, and FEV1 parameters at

the first evaluation (p> 0.05), a statistically significant negative correlation was observed with BMI (r=

-0.37, p= 0.02).

When the patients were grouped according to sex (male and female), age (<12 years and >12 years), BMI (<5th percentile and 5-85th percentile), presence of colonization and *Pseudomonas* colonization, CRP levels (<0.5 mg/dL and >0.5 mg/dL), and FEV1 (≥70% mild and <70% moderate-severe disease), changes in

**Table:** 

**Table 2.** Comparison of atherosclerosis risk factors, systemic inflammation markers, pulmonary function tests, and hemodynamic measurements			
	**First Evaluation**	**Second Evaluation**	**p**
**Atherosclerosis risk factors**			
BMI*	16.35 (13.75-24.1)	16.44 (13.85-24.3)	0.680
Glucose, mg/dL&	87.62 ± 15.73	89.29 ± 10.17	0.490
HbA1c, %	5.2 ± 0.54	5.6 ± 0.51	**0.010**
Lipid profile, mg/dL			
Serum cholesterol	123.05±24.71	118.41 ± 22.49	0.070
HDL	41.67±11.07	46.20 ± 12.17	**<0.001**
LDL	61.47 ± 20.63	56.00 ± 20.88	**0.020**
Triglycerides*	96.88 (53.25-130.5)	86.88 (62-101)	0.170
**Systemic inflammation markers**			
CRP, mg/dL*	0.56 (0.10-0.83)	1.28 (0.22-1.81)	**0.020**
IgG	1442.2 ± 634.6	1411.4 ± 509.3	0.640
Spirometry#, %			
FEV1	78.81 ± 26.70	74.93 ± 27.50	**0.010**
FVC	72.81 ± 22.98	70.00 ± 22.31	0.100
FEV1/FVC	106.45 ± 10.73	102.66 ± 14.47	0.050
FEF25-75	84.27 ± 41.53	80.09 ± 44.86	0.150
**Hemodynamic measurements**			
Heart rate (bpm)	94.51 ± 14.02	94.10 ± 17.36	0.870
Peripheral			
SBP (mm/Hg)	107.05 ± 13.24	113.00 ± 13.70	**0.020**
DBP (mm/Hg)	68.27 ± 10.49	68.40 ± 10.17	0.950
PP (mm/Hg)	38.32 ± 14.59	44.59 ± 12.65	0.060
MAP (mm/Hg)	84.00 ± 11.41	88.81 ± 10.11	**0.030**
Central			
SBP (mm/Hg)	100.86 ± 11.70	100.02 ± 11.91	0.710
DBP (mm/Hg)	68.32 ± 10.55	70.32 ± 9.98	0.360
PP (mm/Hg)	32.08 ± 13.12	29.70 ± 8.75	0.380
AP (mm/Hg)	4.70 ± 4.08	5.83 ± 3.95	0.260
AIx, %	13.72 ± 7.63	30.37 ± 12.38	**<0.001**
PWV, m/s	4.73 ± 0.42	4.75 ± 0.50	0.790
BMI: Body mass index, HDL: High-density lipoprotein, LDL: Low-density lipoprotein, CRP: C-reactive protein, IgG: Immunoglobulin G, FEV1; Forced expiratory volume in 1, FVC: Forced vital capacity, FEF25‐75: Forced expiratory flow during the middle half of FVC, SBP: Systolic blood pressure, DBP: Diastolic blood pressure, PP: Pulse pressure, MAP: Mean arterial pressure, AP: Augmented pressure, AIx; Augmentation index, PWV: Pulse wave velocity, SDS: Standard deviation score. *Data presented as median (min-max), &Fasting blood glucose, #n= 33.

AIx was similar in the groups of ages, presence of microorganism colonization and *Pseudomonas aeru- ginosa* colonization. The changes in AIx were greater in groups of females (22.5 ± 13.51% vs. 11.73 ± 12.91%), in patients with with low BMI (n= 26, 20.73 ± 13.57% vs. n= 11, 8.09 ± 11.55%), high

CRP levels (n= 20, 21.75 ± 12.31% vs. n= 17, 11.35

± 14.36%), and moderate-severe disease (n= 10, 25.90 ± 12.73% vs. n= 23, 12.04 ± 13.03%) (Figure

2). Due to the high diversity of genetic mutations in the study group, changes in AIx based on muta- tion-specific grouping could not be analyzed.

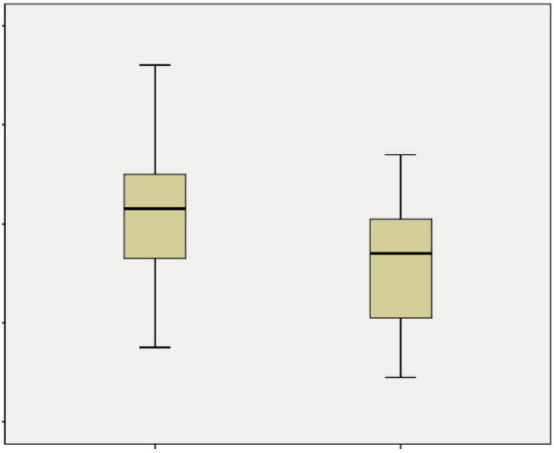

**Figure 2.** Comparison changes in AIx according to CRP levels.

## DISCUSSION

This study showed that arterial stiffness measured with AIx in children with CF increased at follow-up. To our knowledge, this is the first study to evaluate the use of CVD risk markers PWV and AIx in the follow-up of children with CF. At the follow-up, there was a statistically significant increase in HDL, HbA1c, and CRP and a decrease in LDL and FEV1. Peripheral SBP and mean arterial pressure increased statistically significantly. The increase in arterial stiffness (17%) was greater than expected for age and greater in females and in patients with low BMI, moderate-se- vere disease, and high CRP levels. Also, the change in AIx was positively correlated with changes in peripheral SBP and CRP. There was no significant change in heart rate, central BP, and PWV.

Although there are theories about the pathophysiology of CVD in CF, they are still not fully elucidated. This uncertainty creates difficulties in reaching a consen- sus on when and how CVD should be evaluated in CF (10). Screening for lipid parameters, electrocardi- ography, and echocardiogram have been recom- mended for evaluation (3). Increased arterial stiffness with atherosclerosis and vascular aging is a precursor to CVD. In the early stage of CVD, vascular changes are difficult to recognize by traditional methods such as echocardiography (12,13). Therefore, evaluating and monitoring arterial stiffness in CF patients may help to obtain detailed information on the develop- ment and follow-up of cardiovascular events. Our main aim in this study was not to detect CVD, but to

create pioneering research in terms of alternative methods that can be used in children’s evaluating and following CVD risk in these patients and to give ideas for new follow-up strategies.

Therefore, in this study, AIx and PWV measurements were repeated after a median of 14 (12-16) months in children with CF to evaluate the association with CF-specific CVD risk factors at follow-up, and a sta- tistically significant increase was found in AIx. Increased arterial stiffness results in changes in PWV, which measures the speed at which the pressure waveform is transmitted through the large vessels, and in AIx, a measure of the interaction of the for- ward and backward traveling pressure wave arriving at the central arteries (14,15). Changes in aortic PWV reflect central arterial stiffness and are more pro- nounced in individuals older than 50 years. AIx is an indicator of peripheral reflection and is influenced by both macrovascular and microvascular functions. AIx is also related to age, heart rate, BP, ejection time, and peripheral vascular tone. Besides this, epidemio- logical studies have documented a linear relation with age for peripheral and central SBP and central pulse pressure (16). In the current study, the statisti- cally significant difference in peripheral systolic pressure and AIx, but not in central blood pressures and PWV in children with CF at follow-up was thought to be a reflection of the findings of the early stages of arterial stiffness. The increase in AIx and its correlation with an increase in CRP and peripheral SBP may be explained by an increase in sympathetic tone and/or vasoconstrictor tone in smooth muscle through changes in nitric oxide (NO) metabolism due to factors such as systemic inflammation, oxidative stress, and CFTR dysfunction which are possible risk factors in CF.

Recent studies have shown that inflammation is a risk factor for the development of atherosclerosis and CVD even in the pediatric population (17-20). Therefore, the idea that the CF population character- ized by organ-specific and systemic inflammation is at risk for the development of CVD is widespread but has not been fully substantiated by studies. In addi- tion to the recurrent respiratory infections in CF, CFTR dysfunction may contribute to local and sys- temic inflammation. Studies have suggested that CFTR protein is present in both bronchial and pulmo- nary arteries and its dysfunction causes cytokine production, leukocyte infiltration, and inflammation in the pulmonary system (21-23). In addition, CFTR

dysfunction affects calcium concentration in smooth muscle cells, impairs the anti-inflammatory and anti-oxidant functions of NO, and activates leukocyte infiltration by increasing interleukin-8 production

(24). Studies in adults have shown that CRP is higher in CF patients compared to healthy controls and that this is associated with poor clinical outcomes and lower pulmonary function (25,26). In our study, the significant increase in CRP levels, the positive cor- relation of this increase with the increase in AIx, and the fact that the change in AIx was greater in patients with higher CRP levels suggest that inflammation in CF may be an important factor for CVD risk in this disease, which is consistent with studies in the litera- ture and the proposed consensus (3,5,27,28).

Associations between the risk of CVD and disease activity and poor clinical outcomes have been demonstrated in many diseases in both adults and children (29-31). A study on vascular changes follow- ing intravenous antibiotics in adults with CF found a significant reduction in AIx after treatment of pulmo- nary exacerbation and the reduction in AIx after treatment was frequent in patients with poor clinical

condition [baseline FEV1% (r= 0.77, p< 0.01) and BMI (r= 0.71, p< 0.01)] (32). In our previous study,

we measured AIx and PWV before and after treat- ment and one month after the end of treatment in children with acute pulmonary exacerbation. Decreased arterial stiffness (AIx) and CRP and increased spirometry parameters with acute exacer- bation have been demonstrated. Pretreatment AIx is associated with poor clinical outcomes (clinical score, BMI, and PFTs) and systemic inflammation (CRP) (p< 0.05) (33). Ain addition, in this study, the changes in AIx were greater in patients with low BMI and moderate-severe disease and negatively correlat- ed with BMI at first evaluation.

Epidemiological studies have reported that the female sex is associated with poor clinical conditions and higher rates of mortality in CF. In the female sex, clinical conditions such as differences in treatment requirements in pulmonary exacerbations, rapid decline in pulmonary functions, and increased fre- quency of CFRD are thought to be multifactorial and have been suggested to be due to anatomical, bio- chemical such as sex hormones, and physiological differences between the genders (34,35). In our study, consistent with epidemiological studies, we found that the increase in arterial stiffness was greater in females. As is known, AIx is influenced by age, sex,

height, and heart rate (36). It would be appropriate to determine the clinical significance of this result and design studies to increase awareness of the risk of CVD in the female sex with CF.

There were several limitations in this study. The num- ber of patients was small because it was a follow-up study of patients who had previously undergone hemodynamic measurements. The main aim of this study was to examine the changes in arterial stiffness measurements and CF-related risk factors in the fol- low-up of children with CF. Therefore, the mecha- nisms of action that may lead to changes in these arterial stiffness measurements were not evaluated. Furthermore, due to the high genetic diversity of the population in our study, we were unable to compare the change in AIx in mild and severe mutations.

In the present study, we did not specifically evaluate prospective vascular changes specific to CVD condi- tions in CF. We examined changes in arterial stiffness measurements at follow-up in addition to known CVD risk factors. In diseases such as chronic obstruc- tive pulmonary disease, the use of arterial stiffness measurements has been shown to predict cardiovas- cular events and can be used to indicate disease severity independently of classical risk factors (29). The use of arterial stiffness markers in a disease such as CF, which has been ongoing since childhood, may enable early detection and follow-up of the risk of CVD and may enable future measures to be taken. Therefore, using more subclinical markers in CF may help evaluate patients in clinical practice in addition to the diagnostic procedures recommended in guide- lines for CVD.

**Ethical Committee Approval:** This study was approved by Ege University Faculty of Medicine Clinical Research Committee (Decision no: 19-6.1T/40, Date: 28.06.2019).

### CONFLICT of INTEREST

The authors declare that they have no conflict of interest.

